# Measurements of rates of cooling of a manikin insulated with different mountain rescue casualty bags

**DOI:** 10.1186/s13728-017-0055-7

**Published:** 2017-04-20

**Authors:** Christopher Press, Christopher Duffy, Jonathan Williams, Ben Cooper, Neil Chapman

**Affiliations:** 1 Edale Mountain Rescue Team, Hope Cement, Hope Works, Hope, Derbyshire, S33 6RP UK; 2 Department of Anaesthesia, Sheffield Teaching Hospitals NHS Foundation Trust, Northern General Hospital, Herries Road, Sheffield, South Yorkshire S5 7AU UK; 3 The Medical School, The University of Sheffield, Beech Hill Road, Sheffield, South Yorkshire S10 2RX UK; 4 Department of Emergency Medicine, Sheffield Teaching Hospitals NHS Foundation Trust, Northern General Hospital, Herries Road, Sheffield, South Yorkshire S5 7AU UK; 5 The Department of Oncology and Metabolism, Academic Unit of Reproductive and Developmental Medicine, Level 4, The Jessop Wing, The University of Sheffield, Tree Root Walk, Sheffield, South Yorkshire S10 2SF UK

**Keywords:** Hypothermia, Insulation, Pre-hospital, Mountain rescue, Trauma

## Abstract

**Background:**

Accidental hypothermia is common in those who sustain injuries in remote environments. This is unpleasant and associated with adverse effects on subsequent patient outcomes. To minimise further heat loss, a range of insulating systems are available to mountain rescue teams although the most effective and cost-efficient have yet to be determined.

**Methods:**

Under ambient, still, dry, air conditions, a thermal manikin was filled with water at a temperature of 42 °C and then placed into a given insulation system. Water temperature was then continuously observed via an in-dwelling temperature sensor linked to a PROPAQ 100 series monitor and recorded every 10 min for 130 min. This method was repeated for each insulating package.

**Results:**

The vacuum mattress/Pertex©/fibrepile blanket system, either on its own or coupled with the Wiggy bag, was the most efficient with water temperatures only decreasing by 3.2 °C over 130 min. This was followed by the heavy-weight casualty bags without the vacuum mattress/Pertex©/fibrepile blanket system, decreasing by 4.2–4.3 °C. With the Blizzard bag, a decline in water temperature of 5.4 °C was seen over the study duration while a decrease of 9.5 °C was noted when the plastic survival bag was employed.

**Conclusions:**

Under the still-air conditions of the study, the vacuum mattress/Pertex©/fibrepile blanket was seen to offer comparable insulation effectiveness compared to be both heavy-weight casualty bags. In turn, these three systems appeared more efficient at insulating the manikin than the Blizzard bag or plastic survival bag.

**Electronic supplementary material:**

The online version of this article (doi:10.1186/s13728-017-0055-7) contains supplementary material, which is available to authorised users.

## Background

Accidental hypothermia, which can occur at any time of the year, is defined as a core body temperature of <35 °C [1, 2]. At the simplest level, hypothermia can develop in any situation where the equilibrium between heat loss and heat generation favours heat loss. Adverse wilderness environments, particularly those including cold, wet, windy conditions, provide the required parameters where heat loss, through radiation, convection and conduction has the potential to quickly outstrip any residual thermogenic capability of an individual. Indeed, in the UK, pioneering studies by Griffith Pugh into the Peak District-based Four Inns disaster, illustrated that such extreme weather conditions, coupled with ineffective clothing and walking to the point of exhaustion, were the main precipitating features of the fatalities [3–6].

Accidental hypothermia also has a profound negative effect on those physiological survival mechanisms activated in response to trauma—for example, coagulopathies which prolong times to achieve effective haemostasis ([7–9]; reviewed in [10]). In this context, hypothermia is a key component of the so-called “lethal triad”, together with coagulopathy and lactic acidosis, which, when combined in a traumatically injured casualty, work synergistically to greatly reduce the likelihood of survival [11].

The figures for true mountain hypothermia are difficult to find [12]. In Scotland, Hearns’ study [13] identified that 14% of casualties were suffering some form of cold injury upon arrival of the mountain rescue team although this was associated with only one fatality. A similar picture was observed for England and Wales where, in 2011, mountain rescue teams responded to 1074 incidents of which 850 casualties needed medical intervention [14]. Only 27 (~3%) were reported as hypothermic and six (~1%) as exhausted. It is likely, however, that these numbers under-represent the actual incidence of accidental hypothermia given that milder forms may be un-recorded in the other mountain casualties who were, presumably, immobile due to their injuries (e.g. leg injures) and so not able to generate significant heat to maintain core temperature.

**Fig. 1:**
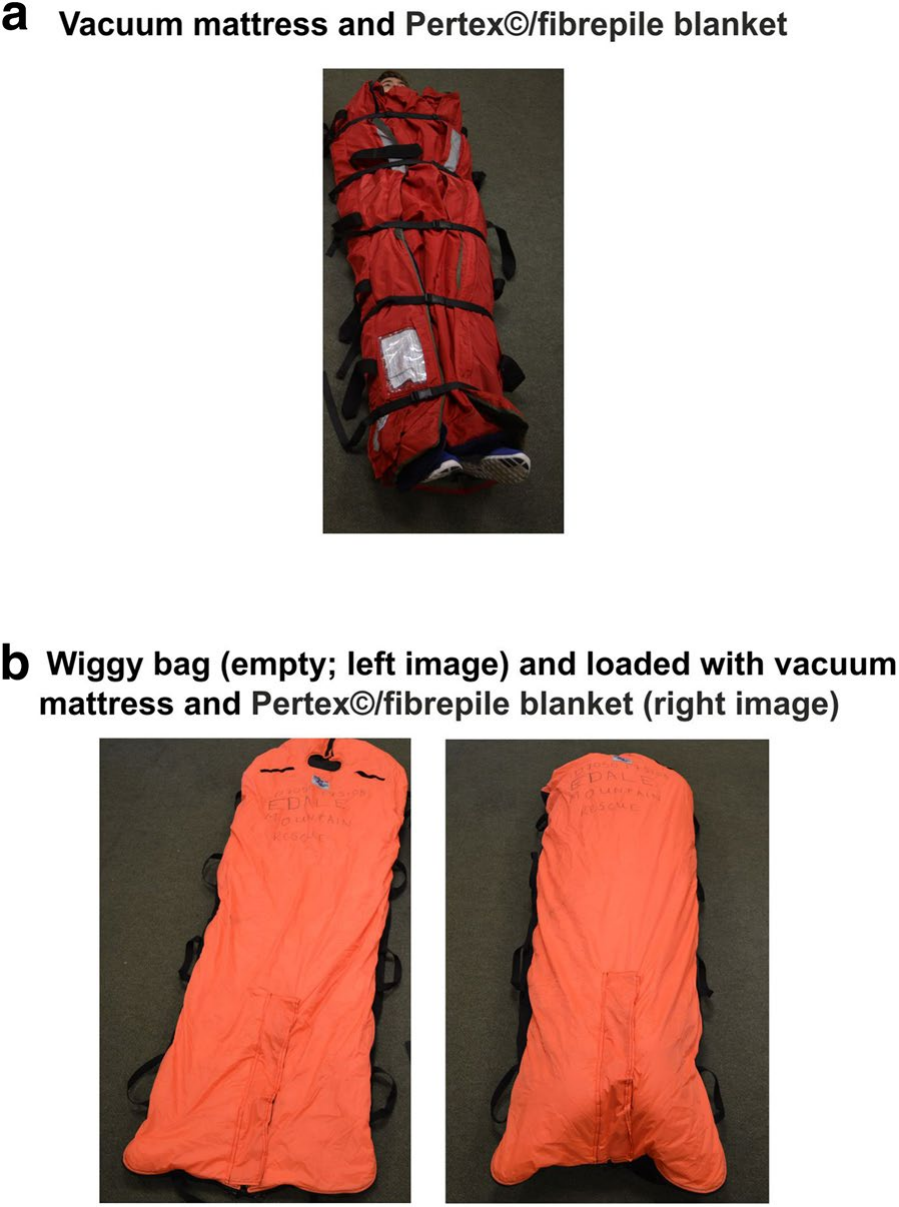
Standard insulation system utilised by Edale Mountain Rescue Team. The casualty is placed in the vacuum mattress and wrapped in a Pertex©/pile blanket (**a**) This can be further supplemented by placing this system within the heavier-weight casualty “Wiggy” bag (**b** *left-hand image*—empty Wiggy bag; *Right-hand image*—Wiggy bag loaded with casualty enclosed in a vacuum mattress/Pertex©/pile blanket)

Within a mountain rescue setting, it is difficult to assess core temperature accurately even with tympanic thermometers [15–17]. Consequently, treatment of accidental hypothermia is generally focused on minimising any further heat loss from the casualty by wrapping them in water-proof/wind-proof thermal bags of which there are many varieties [18–24]. The location of the definitive treatment centre is also considered [16]. Where appropriate, Edale Mountain Rescue Team (Edale MRT) evacuates a casualty by placing the patient in a full body splint (a vacuum mattress) wrapped in a Pertex©/fibrepile blanket (polyester fleece/polyamide water-resistant cover; Fig. 1a, right-hand image). If required, greater insulation and protection from poor weather or winter conditions can be obtained by placing the vacuum mattress and sheet into a heavy duty casualty (Wiggy) bag composed of an inner section of a thicker grade fibrepile and an outer layer of heavy duty water-proof nylon (Fig. 1a, b). Given that there is no optimised protocol for rewarming a hypothermic pre-hospital casualty, we wanted to test the insulating effectiveness of such materials against the system currently employed within our rescue team. Our study investigates the rate of heat loss over a defined time period from a 32 l volume of water placed within a simple thermal manikin using various insulation devices carried by mountain rescue teams (MRTs) or the air ambulance service.

## Methods

**Fig. 2:**
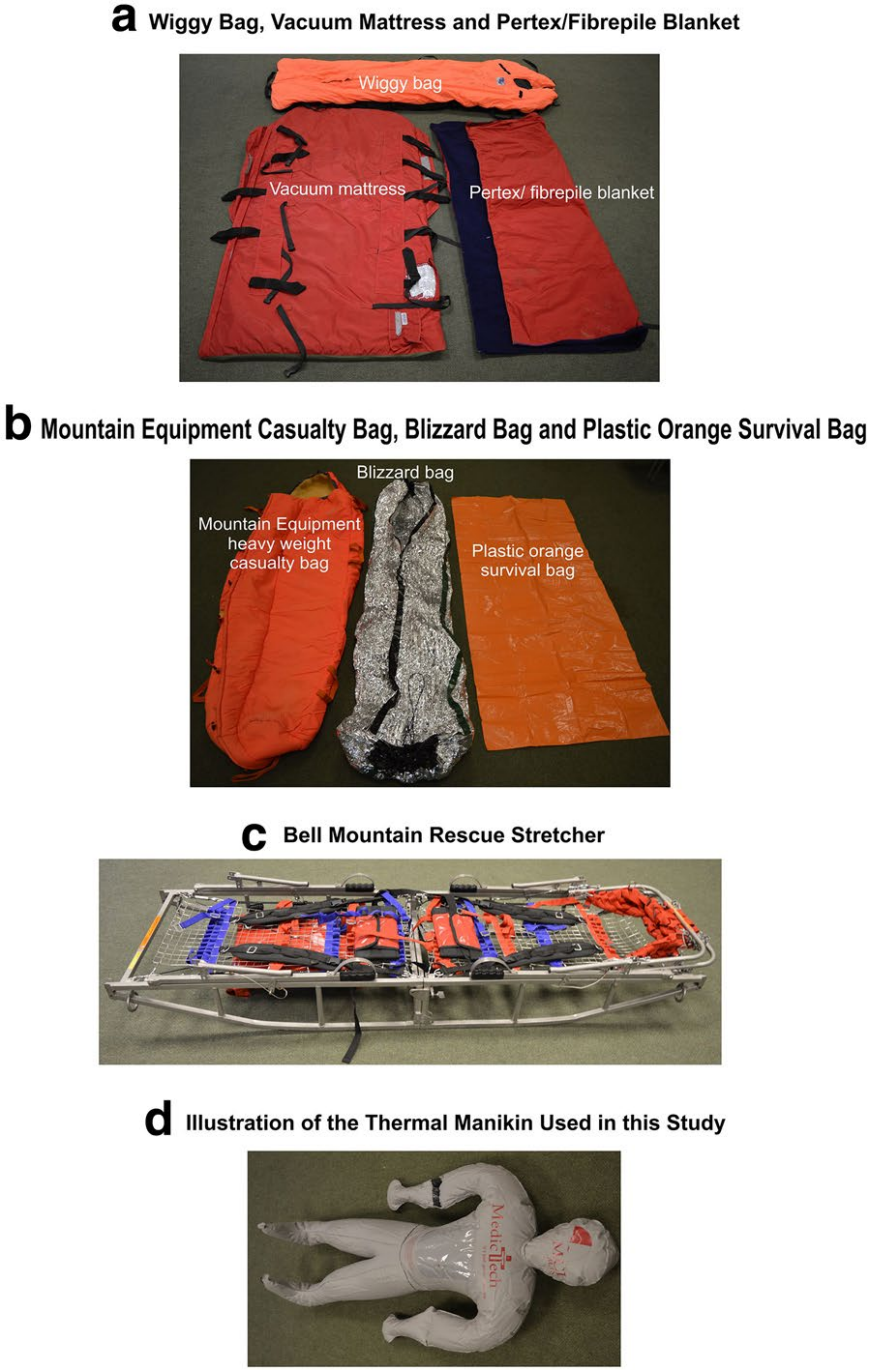
Equipment and insulation systems utilised in this study. Heavy-weight casualty “Wiggy” bag, vacuum mattress and Pertex©/pile blanket (**a**). Mountain equipment heavy-weight casualty bag, Blizzard bag and plastic orange survival bag (**b**). Bell mountain rescue stretcher routinely used by Edale MRT (**c**). The MCI thermal manikin (**d**)

This study was performed within the vehicle bay of Edale MRT Headquarters. The site was chosen due to its generally stable ambient air conditions where the ambient air temperature range was 11–13 °C over the course of the work. Insulation systems were as follows: Casualty Hypothermia Bag (Wiggy Bag; Wiggie’s Inc., Grand Junction, Colorado USA), a vacuum mattress/Pertex© fibrepile blanket system (Snowsled Products, UK) (Fig. 2a); Mountain Equipment (ME) Casualty Bag (Mountain Equipment, Glossop, UK), Blizzard bag, a double-skinned, light-weight foil bag (Blizzard Survival, Bethesda, Wales, UK), and standard orange plastic survival bag (essentially a vapour barrier; Gelert; UK) (Fig. 2b). These systems were supported on a Bell Mountain Rescue Stretcher (57 cm wide × 21.5 cm high × 200 cm length; Fig. 2c; Lyon Work and Rescue, Cumbria, UK).

We utilised a thermal manikin (the MCI Man™, MedicTech, Thomas EMS; Fig. 2d), because our study focussed on the insulation properties of given rescue insulation systems not the ability to rewarm a casualty. As such, this model essentially served as a receptacle for warm water representing a similar surface area and shape of a human body of a height of 170 cm that would be wrapped in a given insulating system on a live rescue (Fig. 2d). It had no physiological capacity to generate de novo heat. A previous study of insulating systems was conducted using bags of dialysate [24] and we believe that this supports the use of our chosen model described herein.

The manikin was filled with 30 l of hot water from an Unvented Direct water pointer heater by Heatrae Sadia, Norwich, England, and a further 2 l were added to adjust the water to an initial temperature of approximately 42 °C; at this stage, the manikin was then placed into a given insulation system and placed in a supine position on to a Bell Mountain Rescue Stretcher (Fig. 2c). The in-dwelling temperature sensor, linked to a PROPAQ 100 series monitor, was placed inside the manikin via an access port on the dorsal surface (not shown) and remained in situ throughout each experiment to facilitate continuous monitoring of the water temperature. Temperature values were noted every 10 min for the duration of each experiment (130 min). This 130-min time course was chosen because it represented an accurate reflection of the prolonged stretcher-carry times experienced by some casualties rescued by Edale MRT. Ambient air temperature recordings within the vehicle bay were taken using a standard laboratory alcohol-based thermometer (Fischer Scientific, UK) hanging above the manikin at defined intervals throughout each day of study. The study was conducted over a period of 6 weeks.

## Statistical analyses

Data were analysed using GraphPad Prism version 6.02 software and compared using analysis of variance, ANOVA; statistical significance was verified using Tukey’s multiple comparisons test; a probability of *p* < 0.05 was considered statistically significant. All experiments were performed three times and results are expressed as the mean ± SEM.

## Results

**Fig. 3:**
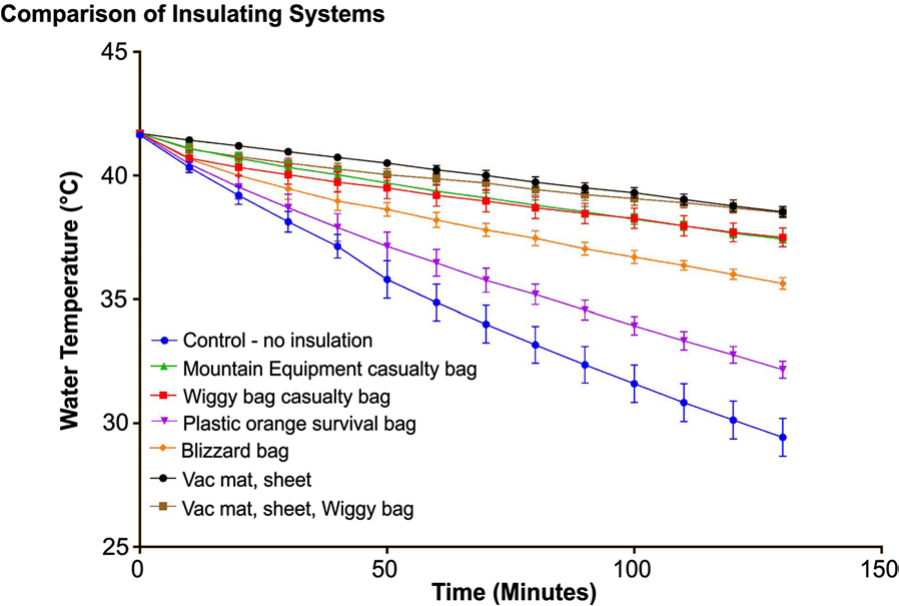
Comparison of insulation system used in mountain rescue. Temperature recordings were taken as described in “Methods” (*n* = 3 for each system). Each system was allowed to equilibrate to the designated start temperature of 42 °C. This was then taken as *t* = 0 min and recordings were subsequently taken every 10 min for 130 min. All experiments were performed three times and data were analysed using ANOVA; statistical significance was verified using Tukey’s multiple comparisons test and results are expressed as the mean ± SEM (*error bars*); *p* < 0.05 was considered statistically significant

This study compared the insulating properties of a number of insulation systems used by pre-hospital agencies including mountain rescue teams. As expected, all insulation devices reduced the rate of cooling of the manikin compared to the control (Fig. 3, compare the six uppermost lines with the control; blue line; Additional file 1: Tables S1–S14). In terms of maintenance of manikin water temperature, the vacuum mattress/Pertex©/fibrepile blanket system, either on its own or coupled with the Wiggy bag, was the most efficient with water temperatures only decreasing by 3.2 °C over 130 min (Table 1). This was followed by the heavy-weight casualty bags without the vacuum mattress/Pertex©/fibrepile blanket system, decreasing by 4.2–4.3 °C (Table 1). With the Blizzard bag, a decline in water temperature of 5.4 °C was seen over the study duration while a decrease of 9.5 °C was noted for the plastic survival bag (Table 1).

**Table 1:** Mean temperature difference of each insulation system tested

Insulation system	Initial mean water temperature (*T*_0_, °C)	Final mean water temperature (*T*_130 min_, °C)	Mean temperature difference (*T*_0_–*T*_130 min_, °C)
Control	41.63	29.43	12.20
Vacuum mattress and Pertex© blanket	41.70	38.53	3.17
Vacuum mattress, Pertex© blanket and Wiggy bag	41.70	38.50	3.20
Wiggy bag	41.70	37.50	4.20
ME casualty bag	41.70	37.43	4.27
Blizzard bag	41.70	35.63	5.37
Plastic orange survival bag	41.70	32.17	9.53

Although not a pre-defined study outcome, the first statistically significant mean temperature differential was observed after 20 min where the un-insulated control was 2 °C cooler than the vacuum mattress/Pertex©/fibrepile blanket combination regularly used by Edale MRT (Fig. 3; blue line vs. black line; Additional file 1: Table S3). Interestingly, we also noted at 20 min that the mean temperature difference between the control and the insulation system comprising the vacuum mattress/Pertex©/fibrepile blanket and Wiggy bag was 1.5 °C (Fig. 3; blue line vs. brown line; Additional file 1: Table S3) suggesting that the vacuum mattress/Pertex©/fibrepile blanket arrangement may be more efficient at reducing heat loss over the initial phases of the study. At 130 min, however, this difference was only 0.03 °C and therefore not deemed significant.

Over the early time course (*T* = 0 to *T* = 30 min), the Blizzard bag compared favourably to the systems employed by mountain rescue teams (Fig. 3; Additional file 1: Tables S1–S4) but at *T* = 40 min, we noted that the Blizzard bag appeared less efficient than the vacuum mattress/Pertex©/fibrepile blanket system (Fig. 3; Additional file 1: Tables S1–S4). Interestingly, no difference was observed when the Blizzard bag was compared to either the Wiggy bag or ME casualty bag in the absence of the vacuum mattress/Pertex©/fibrepile blanket system. Only in the later stages of the experiment (*T* = 100 min) did we note a significant difference in insulation capacity between the Blizzard bag and both the heavy duty casualty bags (Wiggy bag and ME casualty bag; Fig. 3; Additional file 1: Tables S11–S12). No differences were observed throughout the study between the vacuum mattress/Pertex©/fibrepile blanket arrangement and the vacuum mattress/Pertex©/fibrepile blanket/Wiggy bag package (Fig. 3; Additional file 1: Tables S1–S14).

The standard plastic orange survival bag, often carried by many hillwalkers for use in an emergency in remote countryside, offered little in terms of insulation in this study. After only 20 min, there was a temperature difference of 2.3 °C between the orange plastic survival bag and the vacuum mattress/Pertex©/fibrepile blanket combination (Additional file 1: Table S3; plastic orange survival bag vs. the vacuum mattress/Pertex©/fibrepile blanket combination). Moreover, the orange plastic survival bag compared least favourably to all other devices tested in terms of insulating ability with a higher rate of cooling being observed in this experiment (Table 1; Fig. 3; purple line).

## Discussion

In this study, we have compared the effectiveness of insulation systems employed by emergency services to assist casualties who have developed or are at risk of developing hypothermia. We were initially surprised by how effective the vacuum mattress/Pertex© blanket arrangement was in terms of insulating the manikin from heat loss when compared to the heavy-duty casualty bags that are also routinely used. In terms of the vacuum mattress itself, this apparatus is a full-body vacuum splint filled with many tiny polystyrene beads. In use, most air is removed, with the mattress then conforming to the casualty’s body shape ensuring a precise fit. Together with the Pertex©/fibrepile blanket, this arrangement provides good insulation and is believed to minimise cold air pockets that could potentially serve as a heat sink from the casualty. This works effectively for our casualties during summer weather but, because it does not totally enclose the casualty (Fig. 1a, b), it is routinely combined with the Wiggy bag during colder seasons when prevailing weather is less clement. Consequently, although not investigated in this study, one possible explanation for the effectiveness of the vacuum mattress/Pertex©/fibrepile blanket arrangement, compared to the Wiggy bag or Mountain Equipment casualty bag, is that these larger bags are not close fitting and could potentially have greater volumes of cold air within them initially serving as a local heat sink. Further work, however, would be needed to confirm this notion.

The plastic orange survival bag offered little in terms of insulation in this study. That said, many hill walkers carry such bags or similar nylon shelter tents. Our study only examined the ability of a given system to insulate a water-filled manikin under dry, still-air conditions. It did not consider other potentially useful features of such simple equipment including the fact that, in a casualty still able to generate heat, the plastic survival bag would act as a vapour barrier thereby possibly preventing further wetting and reduce some wind-induced convective heat loss [22, 23]. Indeed, while this paper was being peer-reviewed, an anecdotal account from Cairngorm Mountain Rescue Team, Scotland and reported by the BBC, attributed the survival of two benighted hillwalkers on the blizzard-swept Cairngorm plateau to the use of such a plastic bag [25]. It is likely, however, that other factors, such as the clothing and nutritional status of the casualties, also influenced this outcome.

Within a definitive care setting, the receiving hospital is likely to actively rewarm the casualty [9 and references therein]. For those casualties where hypothermia is compounded by trauma, rewarming will also help facilitate haemostasis [10, 11]. In the context of mountain rescue, a defined strategy for pre-hospital rewarming of casualties has not been published although there are a number of reports, with either human subjects or thermal manikins, which describe the effectiveness of various rescue insulation systems including blankets of various thicknesses, military survival bags, bubble-wrap, foil blankets and mountain rescue bags [17–24]. Interestingly, we did observe similarities between our data and previous studies. Principally, the thicker the insulation, the greater the effect of the insulation system. Furthermore, Henriksson et al. [19] also exposed their experimental setup to both high and low wind speeds highlighted the importance of using a layering system: this is similar to our findings with the vacuum mattress/Pertex©/fibrepile blanket/Wiggy bag arrangement.

In terms of UK-based human data, Grant et al. described work comparing the insulating ability of three casualty bags utilised by Scottish MRTs with human subjects by under-defined conditions of an ambient air temperature at −10 °C and a wind speed of 3 m/s [18]. Core and skin temperatures were recorded for up to 60 min. The authors highlighted that none of the bags were seen to perform particularly well given that 15 of the 33 tests were terminated early because the subject’s core temperature decreased to 35.5 °C. It would have been interesting to repeat that study and include the experiments where the subjects were also pre-wrapped in a vacuum mattress/Pertex© blanket described herein to determine if such a layering system made the tests more tolerable: we believe it would.

It is likely that mountain rescue teams will not be afforded the luxury of the time required to affect a full rewarming protocol on the hillside. The corollary, therefore, is that a pre-warmed casualty bag may be the pragmatic option to reduce further heat loss from the patient. Interestingly, a recent study highlighted the use of a heated blanket (Ready-Heat II™) employed by the UK Helicopter Emergency Medical Service (HEMS; [24]). This system offers a light-weight solution which remains warm (~37.8 °C; manufacturer’s details) for 6 h. This blanket clearly has the potential to overcome the gradual heat loss experienced with even the most efficient casualty bags suggesting that there is merit in trialling this blanket as an adjunct to the insulation systems already employed to determine if such a device is cost-effective in the mountain rescue environment. Table 2 illustrates a simple comparison between mass and cost of each insulation system compared to the Ready-Heat II™ blanket.

**Table 2:** Mass (kg) and initial purchase costs of each insulation system tested compared to the Ready-Heat II™ system

Insulation system	Mass (kg)	Cost (£)
Vacuum mattress and Pertex© blanket	8.5	662.00
Wiggy bag	4.5	250.00
ME Casualty bag	4.0	400.00
Blizzard bag	0.39	33.00
Plastic orange survival bag	0.27	2.99
Twelve-panel Ready-Heat II™ system	0.75	20.00

In this comparison, the vacuum mattress/Pertex©/fibrepile blanket system represents an initial large capital outlay (circa £662) but it will be used on the vast majority of incidents and offers broad functionality to rescue teams (insulation, immobilisation, extrication and some load carriage ability) and hence is an effective piece of kit. In contrast, one twelve-panel Ready-Heat II system is ~£20.00: Edale MRT attends an average of 120 incidents/annum giving an annual cost of circa £2400 for a device that only offers insulation and this may be cost-prohibitive for some teams.

## Study limitations

It is difficult to accurately compare data from previous insulation studies with our work described herein because they have either used human subjects or iterative thermal manikins [17–24]. Essentially, the models are not congruent. The human studies are valuable but only use small numbers of subjects and so could be viewed as being under powered (we fully accept, however, that a pragmatic approach needs to be taken when trying to recruit human subjects). The work describing thermal manikins relies on iterative processes and mathematical modelling based on human physiological data. These manikins, such as TORE used by Henriksson et al. [19, 22], have been validated and are repeatedly reliable [19, 22, 26] in terms of physiological responses. They do not, however, consider the psychological effects of cold injury associated with a reduced core temperature [2, 15].

Our model, a thermal manikin filled with warm water, was not intended to accurately reflect the complex physiological processes governing human thermoregulation or those modelled in predictive thermal manikins [19, 22, 26]. Moreover, our model had no physiological capacity to generate de novo heat. In this context, the simplicity of this setup was sufficient to test the effectiveness of the insulation systems over the given time course of 130 min. This it did well. We would, however, advise caution to readers not to over-interpret our data because it is without doubt that the dry, still-air conditions used herein will have influenced our data given that rain and wind have significant detrimental effects on the thermal insulation of a given material [3–5, 19]. Ideally, the work now needs to be extended to include systems that are wet and exposed to wind.

## Conclusions

To conclude, this small-scale study illustrates that in stable ambient air, the vacuum mattress/Pertex©/fibrepile blanket is an effective insulation system more so than thinner foil or plastic bags. The vacuum mattress system can be further enhanced by wrapping this in a heavier weight casualty bag.

## Additional file

**Additional file 1.** Tables S1–S14.

## References

[1] Zafren K, Giesbrecht GG, Danzl DF, Brugger H, Sagalyn EB, Walpoth B, Weiss EA, Auerbach PS, McIntosh SE, Némethy M, McDevitt M, Dow J, Schoene RB, Rodway GW, Hackett PH, Bennett BL, Grissom CK (2014). Wilderness Medical Society practice guidelines for the out-of-hospital evaluation and treatment of accidental hypothermia. Wilderness Environ Med.

[2] Paal P, Gordon L, Strapazzon G, Maeder MB, Putzer G, Walpoth B, Wanscher M, Brown D, Holzer M, Broessner G, Brugger H (2016). Accidental hypothermia–an update. The content of this review is endorsed by the International Commission for Mountain Emergency Medicine (ICAR MEDCOM). Scand J Trauma Resusc Emerg Med.

[3] Pugh LGC (1964). Deaths from exposure on four inns walking competition. Report to Medical Commission on Accident Prevention. Lancet.

[4] Pugh LGCE (1966). Accidental hypothermia in walkers, climbers, and campers: report to the Medical Commission on Accident Prevention. Br Med J.

[5] Pugh LGCE (1966). Clothing insulation and accidental hypothermia in youth. Nature.

[6] Pugh LGCE (1969). Thermal, metabolic, blood, and circulatory adjustments in prolonged outdoor exercise. Br Med J.

[7] Michelson AD, MacGregor H, Barnard MR, Kestin AS, Rohrer MJ, Valeri CR (1994). Hypothermia-induced reversible platelet dysfunction. Thromb Haemost.

[8] Valeri CR, MacGregor H, Cassidy G, Tinney R, Pompei F (1995). Effects of temperature on bleeding time and clotting time in normal male and female volunteers. Crit Care Med.

[9] Watts DD, Trask A, Soeken K, Perdue P, Dols S, Kaufmann C (1998). Hypothermic coagulopathy in trauma: effect of varying levels of hypothermia on enzyme speed, platelet function, and fibrinolytic activity. J Trauma.

[10] Moffatt SW (2013). Hypothermia in trauma. Emerg Med J.

[11] Lier H, Krep H, Schroeder S, Stuber F (2008). Preconditions of hemostasis in trauma: a review. the influence of acidosis, hypocalcemia, anemia, and hypothermia on functional hemostasis in trauma. J Trauma.

[12] Ellerton JA (2006). Casualty care in mountain rescue.

[13] Hearns S (2003). The Scottish mountain rescue casualty study. Emerg Med J.

[14] Feeney G. Mountain rescue (England and Wales) incident report 2011. ISSN 2046-6315. (http://www.mountain.rescue.org.uk/media-centre/statistics).

[15] Giesbrecht GG (2001). Prehospital treatment of hypothermia. Wilderness Environ Med.

[16] Gordon L, Ellerton JA, Paal P, Peek GJ, Barker J (2014). Severe accidental hypothermia. BMJ.

[17] Ducharme MB, Frim J, Bourdon LB, Giesbrecht GG (1997). Evaluation of infrared tympanic thermometers during normothermia and hypothermia in humans. Ann N Y Acad Sci.

[18] Grant SJ, Dowsett D, Hutchison C, Newell J, Connor T, Grant P, Watt M (2002). A comparison of mountain rescue casualty bags in a cold windy environment. Wilderness Environ Med.

[19] Henriksson O, Lundgren PJ, Kuklane K, Holmer I, Bjornstig U (2009). Protection against cold in prehospital care—thermal insulation properties of blankets and rescue bags in different wind conditions. Prehosp Disaster Med.

[20] Lundgren JP, Henriksson O, Pretorius T, BKin, Gerald Bristow G, Chochinov A, Pretorius A, Bjornstig U, Giesbrecht GG (2009). Field torso-warming modalities: a comparative study using a human model. Prehosp Emerg Care.

[21] Thomassen Ø, Færevik H, Østerås Ø, Sunde GA, Zakariassen E, Sandsund M, Heltne JK, Brattebø G. Comparison of three different prehospital wrapping methods for preventing hypothermia—a crossover study in humans. 2011;19:41. http://www.sjtrem.com/content/19/1/41. 10.1186/1757-7241-19-41 PMC3142217 21699720

[22] Henriksson O, Lundgren PJ, Kuklane K, Holmer I, Naredi P, Bjornstig U (2012). Protection against cold in prehospital care: evaporative heat loss reduction by wet clothing removal or the addition of a vapor barrier—a thermal manikin study. Prehosp Disaster Med.

[23] Henriksson O, Lundgren PJ, Kuklane K, Holmer I, Giesbrecht GG, Naredi P, Bjornstig U (2015). Protection against cold in prehospital care: wet clothing removal or addition of a vapor barrier. Wilderness Environ Med.

[24] Zasa M, Flowers N, Zideman D, Hodgetts TJ, Harris T (2016). A torso model comparison of temperature preservation devices for use in the prehospital environment. Emerg Med J.

[25] http://www.bbc.co.uk/news/uk-scotland-highlands-islands-38488123. Accessed 18 Jan 2017.

[26] Psikuta A, Kuklane K, Bogdan A, Havenith G, Annaheim S, Rossi RM (2016). Opportunities and constraints of presently used thermal manikins for thermo-physiological simulation of the human body. Int J Biometeorol.

