# Supplementary material for: Measurements of rates of cooling of a manikin insulated with different mountain rescue casualty bags
Source: Extrem Physiol Med. 2017 Apr 20;6:1. doi: 10.1186/s13728-017-0055-7 (PMC5437540; doi:10.1186/s13728-017-0055-7)
Supplement: Supplementary file 1 — Additional file 1. Tables S1–S14. [file 13728_2017_55_MOESM1_ESM.docx]

| **Supplementary Table 1: T = 0 minutes** | | | | |
| --- | --- | --- | --- | --- |
| **Tukey's multiple comparisons test** | **Mean Diff.** | **95% CI of diff.** | **Significant?** | **Summary** |
|  |  |  |  |  |
|  |  |  |  |  |
| Control vs. Wiggy Bag | -0.06667 | -1.630 to 1.496 | No | ns |
| Control vs. ME Casualty Bag | -0.06667 | -1.630 to 1.496 | No | ns |
| Control vs. Orange Survival bag | -0.06667 | -1.630 to 1.496 | No | ns |
| Control vs. Blizzard Bag | -0.06667 | -1.630 to 1.496 | No | ns |
| Control vs. Vac Mat and Sheet | -0.06667 | -1.630 to 1.496 | No | ns |
| Control vs. Vac Mat, Sheet and Wiggy Bag | -0.06667 | -1.630 to 1.496 | No | ns |
| Wiggy Bag vs. ME Casualty Bag | 0.0 | -1.563 to 1.563 | No | ns |
| Wiggy Bag vs. Plastic Orange Survival bag | 0.0 | -1.563 to 1.563 | No | ns |
| Wiggy Bag vs. Blizzard Bag | 0.0 | -1.563 to 1.563 | No | ns |
| Wiggy Bag vs. Vac Mat and Sheet | 0.0 | -1.563 to 1.563 | No | ns |
| Wiggy Bag vs. Vac Mat, Sheet and Wiggy Bag | 0.0 | -1.563 to 1.563 | No | ns |
| ME Casualty Bag vs. Orange Survival bag | 0.0 | -1.563 to 1.563 | No | ns |
| ME Casualty Bag vs. Blizzard Bag | 0.0 | -1.563 to 1.563 | No | ns |
| ME Casualty Bag vs. Vac Mat and Sheet | 0.0 | -1.563 to 1.563 | No | ns |
| ME Casualty Bag vs. Vac Mat, Sheet and Wiggy Bag | 0.0 | -1.563 to 1.563 | No | ns |
| Orange Survival bag vs. Blizzard Bag | 0.0 | -1.563 to 1.563 | No | ns |
| Orange Survival bag vs. Vac Mat and Sheet | 0.0 | -1.563 to 1.563 | No | ns |
| Orange Survival bag vs. Vac Mat, Sheet and Wiggy Bag | 0.0 | -1.563 to 1.563 | No | ns |
| Blizzard Bag vs. Vac Mat and Sheet | 0.0 | -1.563 to 1.563 | No | ns |
| Blizzard Bag vs. Vac Mat, Sheet and Wiggy Bag | 0.0 | -1.563 to 1.563 | No | ns |
| Vac Mat and Sheet vs. Vac Mat, Sheet and Wiggy Bag | 0.0 | -1.563 to 1.563 | No | ns |

| **Supplementary Table 2: T = 10 minutes** | | | | |
| --- | --- | --- | --- | --- |
| **Tukey's multiple comparisons test** | **Mean Diff.** | **95% CI of diff.** | **Significant?** | **Summary** |
| Control vs. Wiggy Bag | -0.3667 | -1.930 to 1.196 | No | ns |
| Control vs. ME Casualty Bag | -0.7667 | -2.330 to 0.7965 | No | ns |
| Control vs. Plastic Orange Survival bag | -0.1333 | -1.696 to 1.430 | No | ns |
| Control vs. Blizzard Bag | -0.3333 | -1.896 to 1.230 | No | ns |
| Control vs. Vacuum Mattress and Sheet | -1.100 | -2.663 to 0.4631 | No | ns |
| Control vs. Vac Mat, Sheet and Wiggy Bag | -0.7333 | -2.296 to 0.8298 | No | ns |
| Wiggy Bag vs. ME Casualty Bag | -0.4000 | -1.963 to 1.163 | No | ns |
| Wiggy Bag vs. Plastic Orange Survival bag | 0.2333 | -1.330 to 1.796 | No | ns |
| Wiggy Bag vs. Blizzard Bag | 0.03333 | -1.530 to 1.596 | No | ns |
| Wiggy Bag vs. Vacuum Mattress and Sheet | -0.7333 | -2.296 to 0.8298 | No | ns |
| Wiggy Bag vs. Vac Mat, Sheet and Wiggy Bag | -0.3667 | -1.930 to 1.196 | No | ns |
| ME Casualty Bag vs. Plastic Orange Survival bag | 0.6333 | -0.9298 to 2.196 | No | ns |
| ME Casualty Bag vs. Blizzard Bag | 0.4333 | -1.130 to 1.996 | No | ns |
| ME Casualty Bag vs. Vacuum Mattress and Sheet | -0.3333 | -1.896 to 1.230 | No | ns |
| ME Casualty Bag vs. Vac Mat, Sheet and Wiggy Bag | 0.03333 | -1.530 to 1.596 | No | ns |
| Plastic Orange Survival bag vs. Blizzard Bag | -0.2000 | -1.763 to 1.363 | No | ns |
| Plastic Orange Survival bag vs. Vacuum Mattress and Sheet | -0.9667 | -2.530 to 0.5965 | No | ns |
| Plastic Orange Survival bag vs. Vac Mat, Sheet and Wiggy Bag | -0.6000 | -2.163 to 0.9631 | No | ns |
| Blizzard Bag vs. Vacuum Mattress and Sheet | -0.7667 | -2.330 to 0.7965 | No | ns |
| Blizzard Bag vs. Vac Mat, Sheet and Wiggy Bag | -0.4000 | -1.963 to 1.163 | No | ns |
| Vacuum Mattress and Sheet vs. Vac Mat, Sheet and Wiggy Bag | 0.3667 | -1.196 to 1.930 | No | ns |

| **Supplementary Table 3: T = 20 minutes** | | | | |
| --- | --- | --- | --- | --- |
| **Tukey's multiple comparisons test** | **Mean Diff.** | **95% CI of diff.** | **Significant?** | **Summary** |
| Control vs. Wiggy Bag | -1.133 | -2.696 to 0.4298 | No | ns |
| Control vs. ME Casualty Bag | -1.500 | -3.063 to 0.06312 | No | ns |
| Control vs. Plastic Orange Survival bag | -0.3333 | -1.896 to 1.230 | No | ns |
| Control vs. Blizzard Bag | -0.8000 | -2.363 to 0.7631 | No | ns |
| Control vs. Vacuum Mattress and Sheet | -2.000 | -3.563 to -0.4369 | Yes | ** |
| Control vs. Vac Mat, Sheet and Wiggy Bag | -1.567 | -3.130 to -0.003545 | Yes | * |
| Wiggy Bag vs. ME Casualty Bag | -0.3667 | -1.930 to 1.196 | No | ns |
| Wiggy Bag vs. Plastic Orange Survival bag | 0.8000 | -0.7631 to 2.363 | No | ns |
| Wiggy Bag vs. Blizzard Bag | 0.3333 | -1.230 to 1.896 | No | ns |
| Wiggy Bag vs. Vacuum Mattress and Sheet | -0.8667 | -2.430 to 0.6965 | No | ns |
| Wiggy Bag vs. Vac Mat, Sheet and Wiggy Bag | -0.4333 | -1.996 to 1.130 | No | ns |
| ME Casualty Bag vs. Plastic Orange Survival bag | 1.167 | -0.3965 to 2.730 | No | ns |
| ME Casualty Bag vs. Blizzard Bag | 0.7000 | -0.8631 to 2.263 | No | ns |
| ME Casualty Bag vs. Vacuum Mattress and Sheet | -0.5000 | -2.063 to 1.063 | No | ns |
| ME Casualty Bag vs. Vac Mat, Sheet and Wiggy Bag | -0.06667 | -1.630 to 1.496 | No | ns |
| Plastic Orange Survival bag vs. Blizzard Bag | -0.4667 | -2.030 to 1.096 | No | ns |
| Plastic Orange Survival bag vs. Vacuum Mattress and Sheet | -1.667 | -3.230 to -0.1035 | Yes | * |
| Plastic Orange Survival bag vs. Vac Mat, Sheet and Wiggy Bag | -1.233 | -2.796 to 0.3298 | No | ns |
| Blizzard Bag vs. Vacuum Mattress and Sheet | -1.200 | -2.763 to 0.3631 | No | ns |
| Blizzard Bag vs. Vac Mat, Sheet and Wiggy Bag | -0.7667 | -2.330 to 0.7965 | No | ns |
| Vacuum Mattress and Sheet vs. Vac Mat, Sheet and Wiggy Bag | 0.4333 | -1.130 to 1.996 | No | ns |

| **Supplementary Table 4: T = 30 minutes** | | | | |
| --- | --- | --- | --- | --- |
| **Tukey's multiple comparisons test** | **Mean Diff.** | **95% CI of diff.** | **Significant?** | **Summary** |
| Control vs. Wiggy Bag | -1.900 | -3.463 to -0.3369 | Yes | ** |
| Control vs. ME Casualty Bag | -2.200 | -3.763 to -0.6369 | Yes | *** |
| Control vs. Plastic Orange Survival bag | -0.5667 | -2.130 to 0.9965 | No | ns |
| Control vs. Blizzard Bag | -1.333 | -2.896 to 0.2298 | No | ns |
| Control vs. Vacuum Mattress and Sheet | -2.833 | -4.396 to -1.270 | Yes | **** |
| Control vs. Vac Mat, Sheet and Wiggy Bag | -2.367 | -3.930 to -0.8035 | Yes | *** |
| Wiggy Bag vs. ME Casualty Bag | -0.3000 | -1.863 to 1.263 | No | ns |
| Wiggy Bag vs. Plastic Orange Survival bag | 1.333 | -0.2298 to 2.896 | No | ns |
| Wiggy Bag vs. Blizzard Bag | 0.5667 | -0.9965 to 2.130 | No | ns |
| Wiggy Bag vs. Vacuum Mattress and Sheet | -0.9333 | -2.496 to 0.6298 | No | ns |
| Wiggy Bag vs. Vac Mat, Sheet and Wiggy Bag | -0.4667 | -2.030 to 1.096 | No | ns |
| ME Casualty Bag vs. Plastic Orange Survival bag | 1.633 | 0.07021 to 3.196 | Yes | * |
| ME Casualty Bag vs. Blizzard Bag | 0.8667 | -0.6965 to 2.430 | No | ns |
| ME Casualty Bag vs. Vacuum Mattress and Sheet | -0.6333 | -2.196 to 0.9298 | No | ns |
| ME Casualty Bag vs. Vac Mat, Sheet and Wiggy Bag | -0.1667 | -1.730 to 1.396 | No | ns |
| Plastic Orange Survival bag vs. Blizzard Bag | -0.7667 | -2.330 to 0.7965 | No | ns |
| Plastic Orange Survival bag vs. Vacuum Mattress and Sheet | -2.267 | -3.830 to -0.7035 | Yes | *** |
| Plastic Orange Survival bag vs. Vac Mat, Sheet and Wiggy Bag | -1.800 | -3.363 to -0.2369 | Yes | * |
| Blizzard Bag vs. Vacuum Mattress and Sheet | -1.500 | -3.063 to 0.06312 | No | ns |
| Blizzard Bag vs. Vac Mat, Sheet and Wiggy Bag | -1.033 | -2.596 to 0.5298 | No | ns |
| Vacuum Mattress and Sheet vs. Vac Mat, Sheet and Wiggy Bag | 0.4667 | -1.096 to 2.030 | No | ns |

| **Supplementary Table 5: T = 40 minutes** | | | | |
| --- | --- | --- | --- | --- |
| **Tukey's multiple comparisons test** | **Mean Diff.** | **95% CI of diff.** | **Significant?** | **Summary** |
| Control vs. Wiggy Bag | -2.600 | -4.163 to -1.037 | Yes | **** |
| Control vs. ME Casualty Bag | -2.900 | -4.463 to -1.337 | Yes | **** |
| Control vs. Plastic Orange Survival bag | -0.7667 | -2.330 to 0.7965 | No | ns |
| Control vs. Blizzard Bag | -1.833 | -3.396 to -0.2702 | Yes | * |
| Control vs. Vacuum Mattress and Sheet | -3.600 | -5.163 to -2.037 | Yes | **** |
| Control vs. Vac Mat, Sheet and Wiggy Bag | -3.133 | -4.696 to -1.570 | Yes | **** |
| Wiggy Bag vs. ME Casualty Bag | -0.3000 | -1.863 to 1.263 | No | ns |
| Wiggy Bag vs. Plastic Orange Survival bag | 1.833 | 0.2702 to 3.396 | Yes | * |
| Wiggy Bag vs. Blizzard Bag | 0.7667 | -0.7965 to 2.330 | No | ns |
| Wiggy Bag vs. Vacuum Mattress and Sheet | -1.000 | -2.563 to 0.5631 | No | ns |
| Wiggy Bag vs. Vac Mat, Sheet and Wiggy Bag | -0.5333 | -2.096 to 1.030 | No | ns |
| ME Casualty Bag vs. Plastic Orange Survival bag | 2.133 | 0.5702 to 3.696 | Yes | ** |
| ME Casualty Bag vs. Blizzard Bag | 1.067 | -0.4965 to 2.630 | No | ns |
| ME Casualty Bag vs. Vacuum Mattress and Sheet | -0.7000 | -2.263 to 0.8631 | No | ns |
| ME Casualty Bag vs. Vac Mat, Sheet and Wiggy Bag | -0.2333 | -1.796 to 1.330 | No | ns |
| Plastic Orange Survival bag vs. Blizzard Bag | -1.067 | -2.630 to 0.4965 | No | ns |
| Plastic Orange Survival bag vs. Vacuum Mattress and Sheet | -2.833 | -4.396 to -1.270 | Yes | **** |
| Plastic Orange Survival bag vs. Vac Mat, Sheet and Wiggy Bag | -2.367 | -3.930 to -0.8035 | Yes | *** |
| Blizzard Bag vs. Vacuum Mattress and Sheet | -1.767 | -3.330 to -0.2035 | Yes | * |
| Blizzard Bag vs. Vac Mat, Sheet and Wiggy Bag | -1.300 | -2.863 to 0.2631 | No | ns |
| Vacuum Mattress and Sheet vs. Vac Mat, Sheet and Wiggy Bag | 0.4667 | -1.096 to 2.030 | No | ns |
|  |  |  |  |  |

| **Supplementary Table 6: T = 50 minutes** | | | | |
| --- | --- | --- | --- | --- |
| **Tukey's multiple comparisons test** | **Mean Diff.** | **95% CI of diff.** | **Significant?** | **Summary** |
|  |  |  |  |  |
| Control vs. Wiggy Bag | -3.700 | -5.263 to -2.137 | Yes | **** |
| Control vs. ME Casualty Bag | -3.900 | -5.463 to -2.337 | Yes | **** |
| Control vs. Plastic Orange Survival bag | -1.333 | -2.896 to 0.2298 | No | ns |
| Control vs. Blizzard Bag | -2.833 | -4.396 to -1.270 | Yes | **** |
| Control vs. Vacuum Mattress and Sheet | -4.700 | -6.263 to -3.137 | Yes | **** |
| Control vs. Vac Mat, Sheet and Wiggy Bag | -4.233 | -5.796 to -2.670 | Yes | **** |
| Wiggy Bag vs. ME Casualty Bag | -0.2000 | -1.763 to 1.363 | No | ns |
| Wiggy Bag vs. Plastic Orange Survival bag | 2.367 | 0.8035 to 3.930 | Yes | *** |
| Wiggy Bag vs. Blizzard Bag | 0.8667 | -0.6965 to 2.430 | No | ns |
| Wiggy Bag vs. Vacuum Mattress and Sheet | -1.000 | -2.563 to 0.5631 | No | ns |
| Wiggy Bag vs. Vac Mat, Sheet and Wiggy Bag | -0.5333 | -2.096 to 1.030 | No | ns |
| ME Casualty Bag vs. Plastic Orange Survival bag | 2.567 | 1.004 to 4.130 | Yes | **** |
| ME Casualty Bag vs. Blizzard Bag | 1.067 | -0.4965 to 2.630 | No | ns |
| ME Casualty Bag vs. Vacuum Mattress and Sheet | -0.8000 | -2.363 to 0.7631 | No | ns |
| ME Casualty Bag vs. Vac Mat, Sheet and Wiggy Bag | -0.3333 | -1.896 to 1.230 | No | ns |
| Plastic Orange Survival bag vs. Blizzard Bag | -1.500 | -3.063 to 0.06312 | No | ns |
| Plastic Orange Survival bag vs. Vacuum Mattress and Sheet | -3.367 | -4.930 to -1.804 | Yes | **** |
| Plastic Orange Survival bag vs. Vac Mat, Sheet and Wiggy Bag | -2.900 | -4.463 to -1.337 | Yes | **** |
| Blizzard Bag vs. Vacuum Mattress and Sheet | -1.867 | -3.430 to -0.3035 | Yes | ** |
| Blizzard Bag vs. Vac Mat, Sheet and Wiggy Bag | -1.400 | -2.963 to 0.1631 | No | ns |
| Vacuum Mattress and Sheet vs. Vac Mat, Sheet and Wiggy Bag | 0.4667 | -1.096 to 2.030 | No | ns |
|  |  |  |  |  |

| **Supplementary Table 7: T = 60 minutes** | | | | |
| --- | --- | --- | --- | --- |
| **Tukey's multiple comparisons test** | **Mean Diff.** | **95% CI of diff.** | **Significant?** | **Summary** |
|  |  |  |  |  |
| Control vs. Wiggy Bag | -4.333 | -5.896 to -2.770 | Yes | **** |
| Control vs. ME Casualty Bag | -4.500 | -6.063 to -2.937 | Yes | **** |
| Control vs. Plastic Orange Survival bag | -1.600 | -3.163 to -0.03688 | Yes | * |
| Control vs. Blizzard Bag | -3.333 | -4.896 to -1.770 | Yes | **** |
| Control vs. Vacuum Mattress and Sheet | -5.367 | -6.930 to -3.804 | Yes | **** |
| Control vs. Vac Mat, Sheet and Wiggy Bag | -5.000 | -6.563 to -3.437 | Yes | **** |
| Wiggy Bag vs. ME Casualty Bag | -0.1667 | -1.730 to 1.396 | No | ns |
| Wiggy Bag vs. Plastic Orange Survival bag | 2.733 | 1.170 to 4.296 | Yes | **** |
| Wiggy Bag vs. Blizzard Bag | 1.000 | -0.5631 to 2.563 | No | ns |
| Wiggy Bag vs. Vacuum Mattress and Sheet | -1.033 | -2.596 to 0.5298 | No | ns |
| Wiggy Bag vs. Vac Mat, Sheet and Wiggy Bag | -0.6667 | -2.230 to 0.8965 | No | ns |
| ME Casualty Bag vs. Plastic Orange Survival bag | 2.900 | 1.337 to 4.463 | Yes | **** |
| ME Casualty Bag vs. Blizzard Bag | 1.167 | -0.3965 to 2.730 | No | ns |
| ME Casualty Bag vs. Vacuum Mattress and Sheet | -0.8667 | -2.430 to 0.6965 | No | ns |
| ME Casualty Bag vs. Vac Mat, Sheet and Wiggy Bag | -0.5000 | -2.063 to 1.063 | No | ns |
| Plastic Orange Survival bag vs. Blizzard Bag | -1.733 | -3.296 to -0.1702 | Yes | * |
| Plastic Orange Survival bag vs. Vacuum Mattress and Sheet | -3.767 | -5.330 to -2.204 | Yes | **** |
| Plastic Orange Survival bag vs. Vac Mat, Sheet and Wiggy Bag | -3.400 | -4.963 to -1.837 | Yes | **** |
| Blizzard Bag vs. Vacuum Mattress and Sheet | -2.033 | -3.596 to -0.4702 | Yes | ** |
| Blizzard Bag vs. Vac Mat, Sheet and Wiggy Bag | -1.667 | -3.230 to -0.1035 | Yes | * |
| Vacuum Mattress and Sheet vs. Vac Mat, Sheet and Wiggy Bag | 0.3667 | -1.196 to 1.930 | No | ns |
|  |  |  |  |  |

| **Supplementary Table 8: T = 70 minutes** | | | | |
| --- | --- | --- | --- | --- |
| **Tukey's multiple comparisons test** | **Mean Diff.** | **95% CI of diff.** | **Significant?** | **Summary** |
|  |  |  |  |  |
|  |  |  |  |  |
| Control vs. Wiggy Bag | -4.967 | -6.530 to -3.404 | Yes | **** |
| Control vs. ME Casualty Bag | -5.100 | -6.663 to -3.537 | Yes | **** |
| Control vs. Plastic Orange Survival bag | -1.767 | -3.330 to -0.2035 | Yes | * |
| Control vs. Blizzard Bag | -3.800 | -5.363 to -2.237 | Yes | **** |
| Control vs. Vacuum Mattress and Sheet | -6.000 | -7.563 to -4.437 | Yes | **** |
| Control vs. Vac Mat, Sheet and Wiggy Bag | -5.700 | -7.263 to -4.137 | Yes | **** |
| Wiggy Bag vs. ME Casualty Bag | -0.1333 | -1.696 to 1.430 | No | ns |
| Wiggy Bag vs. Plastic Orange Survival bag | 3.200 | 1.637 to 4.763 | Yes | **** |
| Wiggy Bag vs. Blizzard Bag | 1.167 | -0.3965 to 2.730 | No | ns |
| Wiggy Bag vs. Vacuum Mattress and Sheet | -1.033 | -2.596 to 0.5298 | No | ns |
| Wiggy Bag vs. Vac Mat, Sheet and Wiggy Bag | -0.7333 | -2.296 to 0.8298 | No | ns |
| ME Casualty Bag vs. Plastic Orange Survival bag | 3.333 | 1.770 to 4.896 | Yes | **** |
| ME Casualty Bag vs. Blizzard Bag | 1.300 | -0.2631 to 2.863 | No | ns |
| ME Casualty Bag vs. Vacuum Mattress and Sheet | -0.9000 | -2.463 to 0.6631 | No | ns |
| ME Casualty Bag vs. Vac Mat, Sheet and Wiggy Bag | -0.6000 | -2.163 to 0.9631 | No | ns |
| Plastic Orange Survival bag vs. Blizzard Bag | -2.033 | -3.596 to -0.4702 | Yes | ** |
| Plastic Orange Survival bag vs. Vacuum Mattress and Sheet | -4.233 | -5.796 to -2.670 | Yes | **** |
| Plastic Orange Survival bag vs. Vac Mat, Sheet and Wiggy Bag | -3.933 | -5.496 to -2.370 | Yes | **** |
| Blizzard Bag vs. Vacuum Mattress and Sheet | -2.200 | -3.763 to -0.6369 | Yes | *** |
| Blizzard Bag vs. Vac Mat, Sheet and Wiggy Bag | -1.900 | -3.463 to -0.3369 | Yes | ** |
| Vacuum Mattress and Sheet vs. Vac Mat, Sheet and Wiggy Bag | 0.3000 | -1.263 to 1.863 | No | ns |
|  |  |  |  |  |

| **Supplementary Table 9: T = 80 minutes** | | | | |
| --- | --- | --- | --- | --- |
| **Tukey's multiple comparisons test** | **Mean Diff.** | **95% CI of diff.** | **Significant?** | **Summary** |
|  |  |  |  |  |
|  |  |  |  |  |
| Control vs. Wiggy Bag | -5.533 | -7.096 to -3.970 | Yes | **** |
| Control vs. ME Casualty Bag | -5.633 | -7.196 to -4.070 | Yes | **** |
| Control vs. Plastic Orange Survival bag | -2.033 | -3.596 to -0.4702 | Yes | ** |
| Control vs. Blizzard Bag | -4.300 | -5.863 to -2.737 | Yes | **** |
| Control vs. Vacuum Mattress and Sheet | -6.567 | -8.130 to -5.004 | Yes | **** |
| Control vs. Vac Mat, Sheet and Wiggy Bag | -6.267 | -7.830 to -4.704 | Yes | **** |
| Wiggy Bag vs. ME Casualty Bag | -0.1000 | -1.663 to 1.463 | No | ns |
| Wiggy Bag vs. Plastic Orange Survival bag | 3.500 | 1.937 to 5.063 | Yes | **** |
| Wiggy Bag vs. Blizzard Bag | 1.233 | -0.3298 to 2.796 | No | ns |
| Wiggy Bag vs. Vacuum Mattress and Sheet | -1.033 | -2.596 to 0.5298 | No | ns |
| Wiggy Bag vs. Vac Mat, Sheet and Wiggy Bag | -0.7333 | -2.296 to 0.8298 | No | ns |
| ME Casualty Bag vs. Plastic Orange Survival bag | 3.600 | 2.037 to 5.163 | Yes | **** |
| ME Casualty Bag vs. Blizzard Bag | 1.333 | -0.2298 to 2.896 | No | ns |
| ME Casualty Bag vs. Vacuum Mattress and Sheet | -0.9333 | -2.496 to 0.6298 | No | ns |
| ME Casualty Bag vs. Vac Mat, Sheet and Wiggy Bag | -0.6333 | -2.196 to 0.9298 | No | ns |
| Plastic Orange Survival bag vs. Blizzard Bag | -2.267 | -3.830 to -0.7035 | Yes | *** |
| Plastic Orange Survival bag vs. Vacuum Mattress and Sheet | -4.533 | -6.096 to -2.970 | Yes | **** |
| Plastic Orange Survival bag vs. Vac Mat, Sheet and Wiggy Bag | -4.233 | -5.796 to -2.670 | Yes | **** |
| Blizzard Bag vs. Vacuum Mattress and Sheet | -2.267 | -3.830 to -0.7035 | Yes | *** |
| Blizzard Bag vs. Vac Mat, Sheet and Wiggy Bag | -1.967 | -3.530 to -0.4035 | Yes | ** |
| Vacuum Mattress and Sheet vs. Vac Mat, Sheet and Wiggy Bag | 0.3000 | -1.263 to 1.863 | No | ns |
|  |  |  |  |  |

| **Supplementary Table 10: T = 90 minutes** | | | | |
| --- | --- | --- | --- | --- |
| **Tukey's multiple comparisons test** | **Mean Diff.** | **95% CI of diff.** | **Significant?** | **Summary** |
|  |  |  |  |  |
| Control vs. Wiggy Bag | -6.100 | -7.663 to -4.537 | Yes | **** |
| Control vs. ME Casualty Bag | -6.167 | -7.730 to -4.604 | Yes | **** |
| Control vs. Plastic Orange Survival bag | -2.200 | -3.763 to -0.6369 | Yes | *** |
| Control vs. Blizzard Bag | -4.667 | -6.230 to -3.104 | Yes | **** |
| Control vs. Vacuum Mattress and Sheet | -7.133 | -8.696 to -5.570 | Yes | **** |
| Control vs. Vac Mat, Sheet and Wiggy Bag | -6.867 | -8.430 to -5.304 | Yes | **** |
| Wiggy Bag vs. ME Casualty Bag | -0.06667 | -1.630 to 1.496 | No | ns |
| Wiggy Bag vs. Plastic Orange Survival bag | 3.900 | 2.337 to 5.463 | Yes | **** |
| Wiggy Bag vs. Blizzard Bag | 1.433 | -0.1298 to 2.996 | No | ns |
| Wiggy Bag vs. Vacuum Mattress and Sheet | -1.033 | -2.596 to 0.5298 | No | ns |
| Wiggy Bag vs. Vac Mat, Sheet and Wiggy Bag | -0.7667 | -2.330 to 0.7965 | No | ns |
| ME Casualty Bag vs. Plastic Orange Survival bag | 3.967 | 2.404 to 5.530 | Yes | **** |
| ME Casualty Bag vs. Blizzard Bag | 1.500 | -0.06312 to 3.063 | No | ns |
| ME Casualty Bag vs. Vacuum Mattress and Sheet | -0.9667 | -2.530 to 0.5965 | No | ns |
| ME Casualty Bag vs. Vac Mat, Sheet and Wiggy Bag | -0.7000 | -2.263 to 0.8631 | No | ns |
| Plastic Orange Survival bag vs. Blizzard Bag | -2.467 | -4.030 to -0.9035 | Yes | **** |
| Plastic Orange Survival bag vs. Vacuum Mattress and Sheet | -4.933 | -6.496 to -3.370 | Yes | **** |
| Plastic Orange Survival bag vs. Vac Mat, Sheet and Wiggy Bag | -4.667 | -6.230 to -3.104 | Yes | **** |
| Blizzard Bag vs. Vacuum Mattress and Sheet | -2.467 | -4.030 to -0.9035 | Yes | **** |
| Blizzard Bag vs. Vac Mat, Sheet and Wiggy Bag | -2.200 | -3.763 to -0.6369 | Yes | *** |
| Vacuum Mattress and Sheet vs. Vac Mat, Sheet and Wiggy Bag | 0.2667 | -1.296 to 1.830 | No | ns |
|  |  |  |  |  |

| **Supplementary Table 11: T = 100 minutes** | | | | |
| --- | --- | --- | --- | --- |
| **Tukey's multiple comparisons test** | **Mean Diff.** | **95% CI of diff.** | **Significant?** | **Summary** |
|  |  |  |  |  |
|  |  |  |  |  |
| Control vs. Wiggy Bag | -6.667 | -8.230 to -5.104 | Yes | **** |
| Control vs. ME Casualty Bag | -6.633 | -8.196 to -5.070 | Yes | **** |
| Control vs. Plastic Orange Survival bag | -2.333 | -3.896 to -0.7702 | Yes | *** |
| Control vs. Blizzard Bag | -5.100 | -6.663 to -3.537 | Yes | **** |
| Control vs. Vacuum Mattress and Sheet | -7.700 | -9.263 to -6.137 | Yes | **** |
| Control vs. Vac Mat, Sheet and Wiggy Bag | -7.467 | -9.030 to -5.904 | Yes | **** |
| Wiggy Bag vs. ME Casualty Bag | 0.03333 | -1.530 to 1.596 | No | ns |
| Wiggy Bag vs. Plastic Orange Survival bag | 4.333 | 2.770 to 5.896 | Yes | **** |
| Wiggy Bag vs. Blizzard Bag | 1.567 | 0.003548 to 3.130 | Yes | * |
| Wiggy Bag vs. Vacuum Mattress and Sheet | -1.033 | -2.596 to 0.5298 | No | ns |
| Wiggy Bag vs. Vac Mat, Sheet and Wiggy Bag | -0.8000 | -2.363 to 0.7631 | No | ns |
| ME Casualty Bag vs. Plastic Orange Survival bag | 4.300 | 2.737 to 5.863 | Yes | **** |
| ME Casualty Bag vs. Blizzard Bag | 1.533 | -0.02979 to 3.096 | No | ns |
| ME Casualty Bag vs. Vacuum Mattress and Sheet | -1.067 | -2.630 to 0.4965 | No | ns |
| ME Casualty Bag vs. Vac Mat, Sheet and Wiggy Bag | -0.8333 | -2.396 to 0.7298 | No | ns |
| Plastic Orange Survival bag vs. Blizzard Bag | -2.767 | -4.330 to -1.204 | Yes | **** |
| Plastic Orange Survival bag vs. Vacuum Mattress and Sheet | -5.367 | -6.930 to -3.804 | Yes | **** |
| Plastic Orange Survival bag vs. Vac Mat, Sheet and Wiggy Bag | -5.133 | -6.696 to -3.570 | Yes | **** |
| Blizzard Bag vs. Vacuum Mattress and Sheet | -2.600 | -4.163 to -1.037 | Yes | **** |
| Blizzard Bag vs. Vac Mat, Sheet and Wiggy Bag | -2.367 | -3.930 to -0.8035 | Yes | *** |
| Vacuum Mattress and Sheet vs. Vac Mat, Sheet and Wiggy Bag | 0.2333 | -1.330 to 1.796 | No | ns |
|  |  |  |  |  |

| **Supplementary Table 12: T = 110 minutes** | | | | |
| --- | --- | --- | --- | --- |
| **Tukey's multiple comparisons test** | **Mean Diff.** | **95% CI of diff.** | **Significant?** | **Summary** |
|  |  |  |  |  |
|  |  |  |  |  |
| Control vs. Wiggy Bag | -7.133 | -8.696 to -5.570 | Yes | **** |
| Control vs. ME Casualty Bag | -7.133 | -8.696 to -5.570 | Yes | **** |
| Control vs. Plastic Orange Survival bag | -2.500 | -4.063 to -0.9369 | Yes | **** |
| Control vs. Blizzard Bag | -5.533 | -7.096 to -3.970 | Yes | **** |
| Control vs. Vacuum Mattress and Sheet | -8.200 | -9.763 to -6.637 | Yes | **** |
| Control vs. Vac Mat, Sheet and Wiggy Bag | -8.067 | -9.630 to -6.504 | Yes | **** |
| Wiggy Bag vs. ME Casualty Bag | 0.0 | -1.563 to 1.563 | No | ns |
| Wiggy Bag vs. Plastic Orange Survival bag | 4.633 | 3.070 to 6.196 | Yes | **** |
| Wiggy Bag vs. Blizzard Bag | 1.600 | 0.03688 to 3.163 | Yes | * |
| Wiggy Bag vs. Vacuum Mattress and Sheet | -1.067 | -2.630 to 0.4965 | No | ns |
| Wiggy Bag vs. Vac Mat, Sheet and Wiggy Bag | -0.9333 | -2.496 to 0.6298 | No | ns |
| ME Casualty Bag vs. Plastic Orange Survival bag | 4.633 | 3.070 to 6.196 | Yes | **** |
| ME Casualty Bag vs. Blizzard Bag | 1.600 | 0.03688 to 3.163 | Yes | * |
| ME Casualty Bag vs. Vacuum Mattress and Sheet | -1.067 | -2.630 to 0.4965 | No | ns |
| ME Casualty Bag vs. Vac Mat, Sheet and Wiggy Bag | -0.9333 | -2.496 to 0.6298 | No | ns |
| Plastic Orange Survival bag vs. Blizzard Bag | -3.033 | -4.596 to -1.470 | Yes | **** |
| Plastic Orange Survival bag vs. Vacuum Mattress and Sheet | -5.700 | -7.263 to -4.137 | Yes | **** |
| Plastic Orange Survival bag vs. Vac Mat, Sheet and Wiggy Bag | -5.567 | -7.130 to -4.004 | Yes | **** |
| Blizzard Bag vs. Vacuum Mattress and Sheet | -2.667 | -4.230 to -1.104 | Yes | **** |
| Blizzard Bag vs. Vac Mat, Sheet and Wiggy Bag | -2.533 | -4.096 to -0.9702 | Yes | **** |
| Vacuum Mattress and Sheet vs. Vac Mat, Sheet and Wiggy Bag | 0.1333 | -1.430 to 1.696 | No | ns |
|  |  |  |  |  |

| **Supplementary Table 13: T = 120 minutes** | | | | |
| --- | --- | --- | --- | --- |
| **Tukey's multiple comparisons test** | **Mean Diff.** | **95% CI of diff.** | **Significant?** | **Summary** |
|  |  |  |  |  |
|  |  |  |  |  |
| Control vs. Wiggy Bag | -7.567 | -9.130 to -6.004 | Yes | **** |
| Control vs. ME Casualty Bag | -7.533 | -9.096 to -5.970 | Yes | **** |
| Control vs. Plastic Orange Survival bag | -2.633 | -4.196 to -1.070 | Yes | **** |
| Control vs. Blizzard Bag | -5.867 | -7.430 to -4.304 | Yes | **** |
| Control vs. Vacuum Mattress and Sheet | -8.633 | -10.20 to -7.070 | Yes | **** |
| Control vs. Vac Mat, Sheet and Wiggy Bag | -8.567 | -10.13 to -7.004 | Yes | **** |
| Wiggy Bag vs. ME Casualty Bag | 0.03333 | -1.530 to 1.596 | No | ns |
| Wiggy Bag vs. Plastic Orange Survival bag | 4.933 | 3.370 to 6.496 | Yes | **** |
| Wiggy Bag vs. Blizzard Bag | 1.700 | 0.1369 to 3.263 | Yes | * |
| Wiggy Bag vs. Vacuum Mattress and Sheet | -1.067 | -2.630 to 0.4965 | No | ns |
| Wiggy Bag vs. Vac Mat, Sheet and Wiggy Bag | -1.000 | -2.563 to 0.5631 | No | ns |
| ME Casualty Bag vs. Plastic Orange Survival bag | 4.900 | 3.337 to 6.463 | Yes | **** |
| ME Casualty Bag vs. Blizzard Bag | 1.667 | 0.1035 to 3.230 | Yes | * |
| ME Casualty Bag vs. Vacuum Mattress and Sheet | -1.100 | -2.663 to 0.4631 | No | ns |
| ME Casualty Bag vs. Vac Mat, Sheet and Wiggy Bag | -1.033 | -2.596 to 0.5298 | No | ns |
| Plastic Orange Survival bag vs. Blizzard Bag | -3.233 | -4.796 to -1.670 | Yes | **** |
| Plastic Orange Survival bag vs. Vacuum Mattress and Sheet | -6.000 | -7.563 to -4.437 | Yes | **** |
| Plastic Orange Survival bag vs. Vac Mat, Sheet and Wiggy Bag | -5.933 | -7.496 to -4.370 | Yes | **** |
| Blizzard Bag vs. Vacuum Mattress and Sheet | -2.767 | -4.330 to -1.204 | Yes | **** |
| Blizzard Bag vs. Vac Mat, Sheet and Wiggy Bag | -2.700 | -4.263 to -1.137 | Yes | **** |
| Vacuum Mattress and Sheet vs. Vac Mat, Sheet and Wiggy Bag | 0.06667 | -1.496 to 1.630 | No | ns |
|  |  |  |  |  |

| **Supplementary Table 14: T = 130 minutes** | | | | |
| --- | --- | --- | --- | --- |
| **Tukey's multiple comparisons test** | **Mean Diff.** | **95% CI of diff.** | **Significant?** | **Summary** |
|  |  |  |  |  |
|  |  |  |  |  |
| Control vs. Wiggy Bag | -8.067 | -9.630 to -6.504 | Yes | **** |
| Control vs. ME Casualty Bag | -8.000 | -9.563 to -6.437 | Yes | **** |
| Control vs. Plastic Orange Survival bag | -2.733 | -4.296 to -1.170 | Yes | **** |
| Control vs. Blizzard Bag | -6.200 | -7.763 to -4.637 | Yes | **** |
| Control vs. Vacuum Mattress and Sheet | -9.100 | -10.66 to -7.537 | Yes | **** |
| Control vs. Vac Mat, Sheet and Wiggy Bag | -9.067 | -10.63 to -7.504 | Yes | **** |
| Wiggy Bag vs. ME Casualty Bag | 0.06667 | -1.496 to 1.630 | No | ns |
| Wiggy Bag vs. Plastic Orange Survival bag | 5.333 | 3.770 to 6.896 | Yes | **** |
| Wiggy Bag vs. Blizzard Bag | 1.867 | 0.3035 to 3.430 | Yes | ** |
| Wiggy Bag vs. Vacuum Mattress and Sheet | -1.033 | -2.596 to 0.5298 | No | ns |
| Wiggy Bag vs. Vac Mat, Sheet and Wiggy Bag | -1.000 | -2.563 to 0.5631 | No | ns |
| ME Casualty Bag vs. Plastic Orange Survival bag | 5.267 | 3.704 to 6.830 | Yes | **** |
| ME Casualty Bag vs. Blizzard Bag | 1.800 | 0.2369 to 3.363 | Yes | * |
| ME Casualty Bag vs. Vacuum Mattress and Sheet | -1.100 | -2.663 to 0.4631 | No | ns |
| ME Casualty Bag vs. Vac Mat, Sheet and Wiggy Bag | -1.067 | -2.630 to 0.4965 | No | ns |
| Plastic Orange Survival bag vs. Blizzard Bag | -3.467 | -5.030 to -1.904 | Yes | **** |
| Plastic Orange Survival bag vs. Vacuum Mattress and Sheet | -6.367 | -7.930 to -4.804 | Yes | **** |
| Plastic Orange Survival bag vs. Vac Mat, Sheet and Wiggy Bag | -6.333 | -7.896 to -4.770 | Yes | **** |
| Blizzard Bag vs. Vacuum Mattress and Sheet | -2.900 | -4.463 to -1.337 | Yes | **** |
| Blizzard Bag vs. Vac Mat, Sheet and Wiggy Bag | -2.867 | -4.430 to -1.304 | Yes | **** |
| Vacuum Mattress and Sheet vs. Vac Mat, Sheet and Wiggy Bag | 0.03333 | -1.530 to 1.596 | No | ns |
